# Induction of VCID via hyperhomocysteinemia leads to vision and retina changes in mice

**DOI:** 10.1002/alz.089306

**Published:** 2025-01-03

**Authors:** Erica M. Weekman, Colin B Rogers, Tiffany L. Sudduth, Donna M. Wilcock

**Affiliations:** ^1^ Indiana University School of Medicine/Stark Neurosciences Research Institute, Indianapolis, IN USA; ^2^ University of Kentucky College of Medicine, Sanders‐Brown Center on Aging, Lexington, KY USA; ^3^ University of Kentucky / Sanders‐Brown Center on Aging, Lexington, KY USA; ^4^ Indiana University School of Medicine, Stark Neurosciences Research Institute, Department of Neurology, Indianapolis, IN USA

## Abstract

**Background:**

Diagnosis of Alzheimer’s disease (AD) via MRI is costly and can be limited by regional availability. With the recent advancements and discovery of amyloid in the retina, diagnosis of AD and the effect of AD pathology on the retina is becoming well characterized. However, the prevalence of vascular contributions to cognitive impairment and dementia (VCID) and its effects on the retina are less well known. With the retina being a highly vascularized tissue and the considerable overlap of AD with VCID, it is imperative to understand the effect of VCID on vision.

**Method:**

We placed 6‐month‐old mice on a diet deficient in B vitamins and enriched in methionine to induce hyperhomocysteinemia (HHcy). HHcy is a risk factor for VCID, stroke and AD, and has been well characterized in our lab. After 14 weeks on diet, mice underwent the Visual‐Stimuli 4‐arm Maze (ViS4M) to identify visual and cognitive abnormalities. After behavior, brains and eyes were harvested with the left eye fixed in 4% PFA for 24hrs and the right eye flash frozen for RNA extraction. The fixed retina was flat mounted and stained for vessels, GFAP, and IBA‐1 and the flash frozen retina was used for RNA isolation and NanoString analysis.

**Result:**

Over the seven days the mice were tested on the ViS4M, the mice on the HHcy diet showed impaired cognition and altered colored arm entries compared to control mice. HHcy mice made more 2 arm alternations than 3 or 4 arm alternations, suggesting diminished exploration. When we determined their arm transitions, we saw that the HHcy mice tended to avoid the blue arm, suggesting sensitivity to blue light. In the retina, we saw slightly less vessel volume in the HHcy diet mice along with reduced coverage of vessels by microglia and astrocytes combined.

**Conclusion:**

The high prevalence of VCID with AD along with the impact of AD pathology on the eye makes it critical to understand the effect of VCID on the retina. In our model of HHcy induced VCID, we determined that HHcy does impair both cognition and vision and affects vessels within the retina.

